# Dataset for miRNA expression analysis in the peripheral white blood cells of beef heifers at weaning

**DOI:** 10.1016/j.dib.2023.109515

**Published:** 2023-08-22

**Authors:** Priyanka Banerjee, Wellison J.S. Diniz, Paul W. Dyce

**Affiliations:** Department of Animal Sciences, Auburn University, Auburn, AL 36849, United States

**Keywords:** Fertility, miRNA expression, miRDeep2, Reproductive outcome, Weaning

## Abstract

Subfertility in beef heifers leads to a substantial economic loss for producers and beef industry. To overcome this problem, producers require an efficient system to discriminate beef heifers with varying reproductive potential as early as possible. MicroRNAs are short non-coding RNAs that post-transcriptionally regulate gene expression. Herein, we profiled the miRNAs in peripheral white blood cells (PWBC) of beef heifers at weaning to investigate the differences in the beef heifers with varying reproductive outcomes. Blood samples from Angus-Simmental crossbred heifers were collected at weaning. The blood was processed to extract the PWBC pellet and was stored at -80 °C until further processing. After the synchronization of estrus and breeding protocol (artificial insemination (AI) followed by natural bull service) and pregnancy diagnosis, the heifers were categorized as fertile (pregnant to AI) or subfertile (not pregnant to AI or bull exposure). Total RNA was extracted from PWBC collected at the time of weaning from the fertile and subfertile heifers. After quality assessment, the total RNA was used to prepare libraries. The quality-checked libraries (*n = 14*; 7 samples per fertile and subfertile group) were pooled and sequenced (single-end 50 bp) using a NextSeq 500 platform. The raw sequence reads were analyzed using a bioinformatics workflow utilizing FastQC and MultiQC for quality control, Cutadapt for adapter trimming, miRDeep2 for alignment, and DESeq2 for differential expression analysis. The raw and normalized miRNA counts were deposited and made publicly available on the gene expression omnibus database (GEO; GSE225854). This is the first dataset investigating the miRNA expression level in PWBC at weaning in beef heifers to predict the future reproductive outcome. The results from the data presented here are reported in the research article titled “miRNA expression profiles of peripheral white blood cells from beef heifers with varying reproductive potential” [1].

Specifications Table

**Table:** 

Subject	Agricultural and Biological Sciences
Specific subject area	Animal Science, Omics: Transcriptomics
Type of data	miRNA-Seq raw data (FASTQ format), .text file, .csv file, Figure, and Table
How the data were acquired	Illumina NextSeq 500 using single-end 50 bp chemistry
Data format	Raw miRNA-Seq data (FASTQ format), Raw read counts (.txt format), Normalized counts (.csv format)
Description of data collection	Blood samples were collected and processed to isolate PWBC pellets. Total RNA was extracted from PWBC pellets using Trizol reagent following standard procedures. After RNA quality check, the small RNA-Seq libraries were prepared from the total RNA, and sequencing was performed on the NextSeq 500 platform (Illumina) at the Discovery life sciences (Hudson Alpha Institute of Biotechnology, Huntsville, AL, USA). Single-end 50 bp reads were generated for each sample (*n = 14*).
Data source location	Alabama Research and Extension Center (Auburn University) and Department of Animal Sciences, Auburn University, Auburn, Alabama, USA
Data accessibility	All relevant data (raw and processed miRNA-Seq data) were deposited on: Repository name: Gene Expression Omnibus (GEO) **Data identification number**: GSE225854 **Direct URL to data:** https://www.ncbi.nlm.nih.gov/geo/query/acc.cgi?acc=GSE225854
Related research article	miRNA expression profiles of peripheral white blood cells from beef heifers with varying reproductive potential. Banerjee, P. B.; Diniz, W. J. S.; Rodning, S. P.; Dyce, P. W. Front. Genet. 14 (2023). (https://doi.org/10.3389/fgene.2023.1174145).

## 1 Value of the Data

• This dataset provides the miRNA profile of bovine PWBC collected at weaning with groups representing different reproductive outcomes.
• Since miRNAs regulate the genes, the data can be helpful for researchers interested in studying target genes and pathways underlying heifer fertility.
• The dataset can be compared with miRNA analysis from other cell or tissue types reported and involved in beef heifer fertility.
• The methodology for predicting miRNAs provides a foundation for similar analysis and can be applied to explore the regulatory roles of additional miRNAs of interest.
• The dataset provides the ability to determine the significant differentially expressed miRNAs between the samples with varying future reproductive outcomes. The differentially expressed miRNAs could serve as potential biomarkers for the early identification of a population of subfertile heifers. These miRNAs could be used for a meta-analysis with miRNA profiles generated at different time points, such as during artificial insemination (AI). This will help to understand the trend of miRNAs over time, i.e., from weaning to AI, and identify the candidate genes and altered pathways regulated by these miRNAs that may contribute to contrasting reproductive outcomes.

## 2 Objective

Heifer subfertility is one of the leading contributors to economic loss in the beef industry. Before the breeding season, heifers are selected based on morphological parameters; however, many still fail to conceive. Therefore, molecular candidates that have the potential to discriminate fertile from subfertile beef heifers at weaning, when the replacement heifers are selected, would be very valuable to the beef industry. The current dataset was generated to identify the miRNA expression profile from the PWBC of beef heifers at weaning that were retrospectively classified as fertile or subfertile based on pregnancy results. The objective of generating and using this dataset is to identify miRNAs at different levels and, therefore, the potential candidate genes targeted by these miRNAs at weaning that could predict the future reproductive potential of beef heifers.

## 3 Data Description

**Fig. 1:**
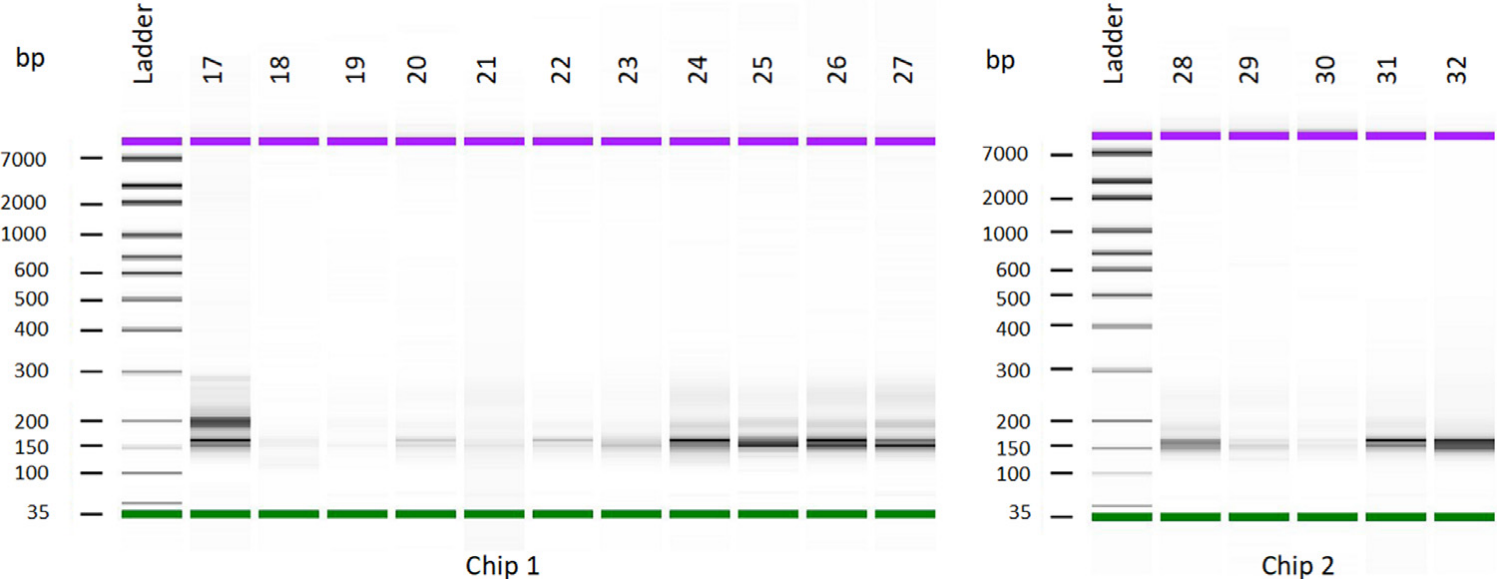
Size distribution of each library constructed checked using Agilent Bioanalyzer high sensitivity DNA assay. The samples were run across two chips for the assay. (For interpretation of the references to color in this figure legend, the reader is referred to the web version of this article.)

**Fig. 2:**
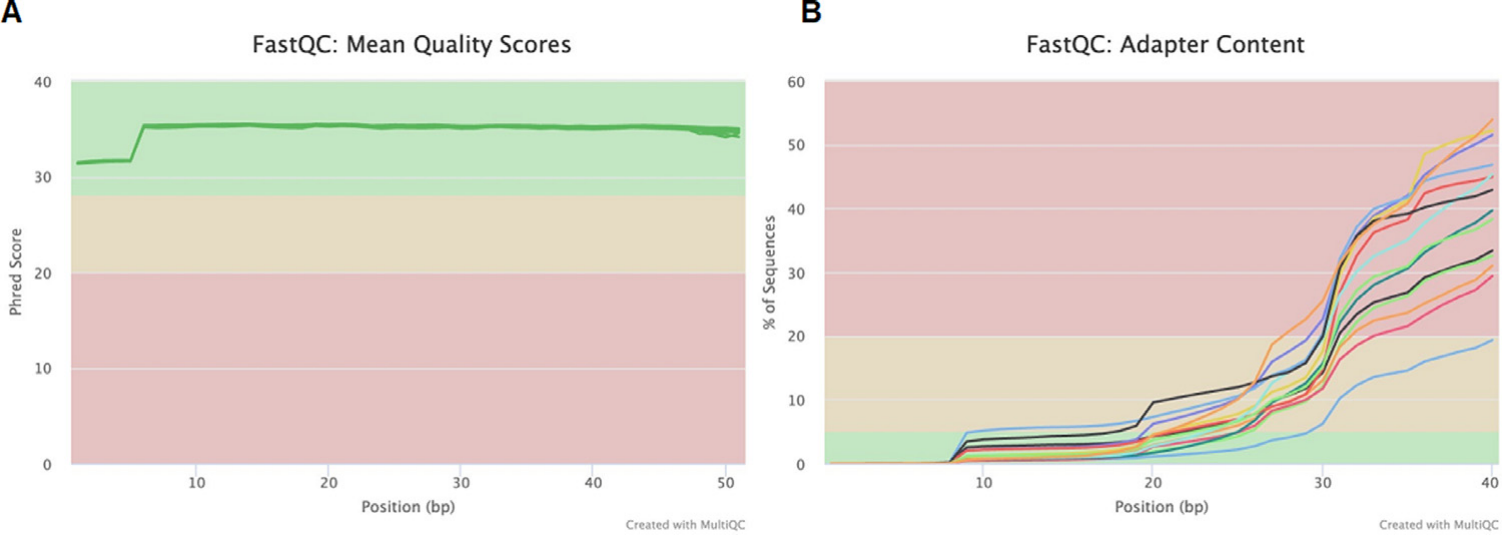
Overview of the raw miRNA-Seq data from peripheral white blood cells (PWBC) of beef heifers at weaning retrospectively classified as pregnant to AI (fertile) or non-pregnant (subfertile). (A) Overall Phred scores of raw FastQ files; (B) Cumulative plot of the fraction of reads where the sequence library adapter sequence is identified in all samples at the indicated base position. (For interpretation of the references to color in this figure legend, the reader is referred to the web version of this article.)

**Fig. 3:**
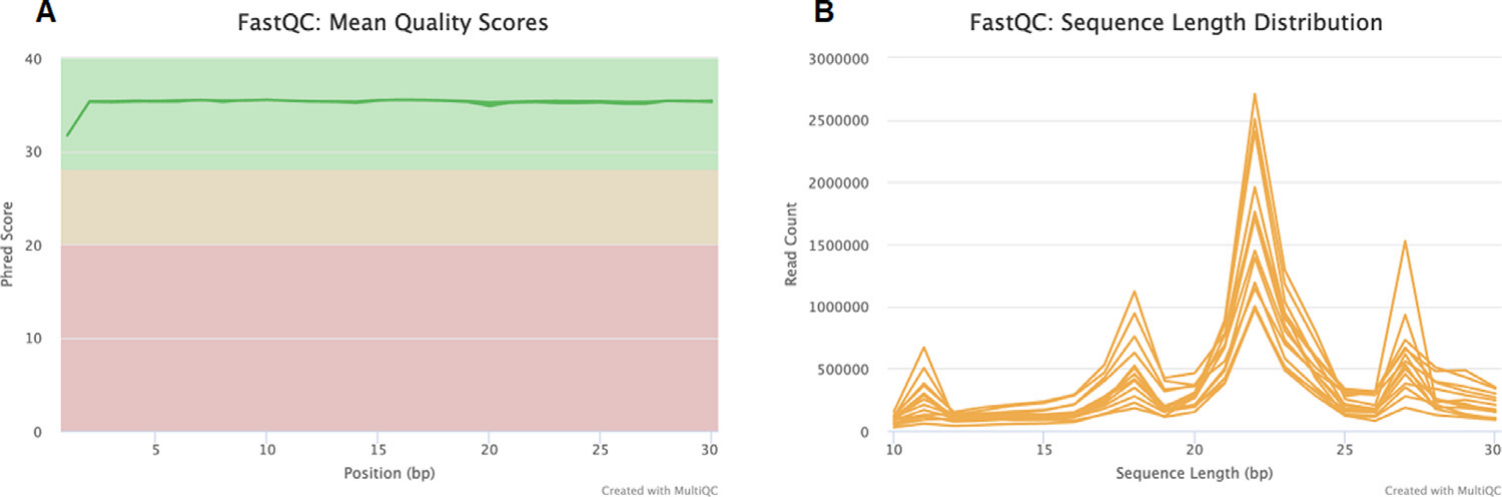
Overview of the trimmed miRNA-Seq data. (A) Overall Phred scores of trimmed FastQ files; (B) miRNA length distribution in all samples with a sharp peak between 20–25 bp. (For interpretation of the references to color in this figure legend, the reader is referred to the web version of this article.)

The miRNA dataset was generated from the PWBC of beef heifers with varying reproductive potential (fertile and subfertile). To generate the data, total RNA extracted from the PWBC (*n = 16*) was used to prepare RNA-Seq libraries in-house using Perkin Elmer NEXTflex small RNA-Seq kit v3. The size distribution of the final libraries was assessed using an Agilent Bioanalyzer high-sensitivity DNA assay, as shown in Fig. 1. Presence of a strong ∼150 bp band as seen in Fig. 1 indicates a successful library preparation (Perkin Elmer). The library preparation was not successful for two samples (18 and 19), exhibited by the absence of a strong band at ∼150 bp. Therefore, they were not considered for sequencing. The remaining libraries (*n = 14*) were pooled and sequenced in NextSeq 500 using single-end 50 bp chemistry to generate raw sequence reads. The raw sequence demultiplexed reads were checked for quality using FastQCv0.11.9 and MultiQCv1.12 (Fig. 2). The raw data was checked for the read quality (Phred score > 30) (Fig. 2A) and adapter content (Fig. 2B). The 3′ adapter sequence was trimmed using Cutadapt. The trimmed Fastq files were checked for quality using FastQC and MultiQC (Fig. 3). The sequencing generated, on average, 6.9 million reads, of which 78.13% of reads were uniquely mapped to the *Bos taurus* reference genome (Table 1). The clean mapped data were subjected to differential expression and functional analysis. The raw reads from 14 samples, the raw gene counts, and the normalized counts are publicly available on the GEO database (GEO accession ID: GSE225854).

**Table 1:** miRNA sequencing summary mapping statistics.

Sample ID	M Seqs	%Mapped	Group
17	4.1	78.781	Subfertile
20	5.1	77.378	Fertile
21	5.9	77.249	Subfertile
22	4.5	72.268	Fertile
23	8.5	77.229	Subfertile
24	5.2	69.145	Fertile
25	9.5	87.118	Fertile
26	7.7	75.899	Subfertile
27	8.6	88.996	Fertile
28	9.4	70.008	Subfertile
29	7.6	86.709	Fertile
30	5.7	88.537	Subfertile
31	6.7	80.708	Fertile
32	8.5	63.795	Subfertile

**Average**	**6.9**	**78.13**	

M Seqs = Million reads sequenced, % Aligned = Percentage of reads aligned to the reference genome, Group = Group to which the sample belongs according to the pregnancy status.

## 4 Experimental Design, Materials and Methods

### 4.1 Animal Handling, Pregnancy Determination, and Heifer Selection

Crossbred Angus-Simmental heifers used in this study were developed and housed at the Alabama Research Center (Auburn University). Blood samples (10 mL) were collected ∼238 days after birth (weaning) in EDTA-coated vacutainer tubes (Becton, Dickinson and Company, Franklin, NJ) and transported to the lab on ice for further processing. At breeding, all heifers followed the same protocol, synchronization of estrus and fixed-time artificial insemination (FTAI), as described previously [2]. Fourteen days following FTAI, the heifers were exposed to fertile bulls for 60 days to ensure adequate opportunities for the beef heifers to conceive. Seventy-five days after AI, pregnancy status was evaluated by transrectal palpation. The heifers were classified as pregnant to AI (fertile), pregnant to natural service, or non-pregnant (subfertile) based on the presence or absence of a conceptus. For the current study, the heifers belonging to fertile and subfertile groups were used.

### 4.2 Sample Collection, Processing, and RNA Extraction

The RNA was extracted from the blood (peripheral white blood cells) collected at the time of weaning. At first, the blood was centrifuged at 1500 x g for 10 min at 4 °C, then the buffy coat was separated and added into a fresh conical tube with 14 mL red blood cell lysis buffer (Cold Spring Harbor Protocols) and incubated for 10 min at room temperature. The tubes were centrifuged at 500 x g for 5 min at 4 °C to pellet the PWBC, followed by a PBS/ 2% fetal bovine serum (FBS) wash. After discarding the supernatant, the clean PWBC pellet was stored at – 80 °C until further processing. For extracting total RNA from the PWBC, Trizol extraction (Invitrogen, Carlsbad, CA, USA) followed by a DNase digestion step was used. Total RNA quality and RNA integrity were assessed using an Agilent Bioanalyzer with the Agilent RNA 6000 Nano kit (Agilent, Santa Clara, CA, USA). The samples with an average RNA integrity number (RIN) > 6.8 were further processed for library construction.

### 4.3 Library Preparation and Sequencing

Total RNA from each sample was diluted with RNase-free water to obtain a final amount of 1 μg as a starting material for library construction. The libraries were prepared using the standard protocols from NEXTflex small RNA-Seq kit v3 (Perkin Elmer). The 5′ and 3′ adapters were ligated to the RNA fragments, which were reverse transcribed and amplified (18 cycles) to generate cDNA libraries using a unique barcode primer. The libraries were cleaned using NEXTflex Cleanup beads (gel-free protocol). The size distribution for the final library was assessed by Agilent Bioanalyzer high-sensitivity DNA assay (Agilent, Santa Clara, CA, United States). The quality-checked libraries were sequenced using NextSeq 500 at Discovery Life Sciences (Hudson Alpha Institute of Biotechnology, Huntsville, AL, United States). Single-end 50 bp data was generated for each sample.

### 4.4 Data Analysis

After sequencing, the quality of raw de-multiplexed reads was performed using FastQC v0.11.9 [3] and MultiQC v1.12 [4]. The 3′ adapters were trimmed using Cutadapt [5] with the following parameters: *-a* TGGAATTCTCGGGTGCCAAGG -*minimum length 23*, followed by removal of four bases from either side of each read (cutadapt -u 4 -u −4) (recommended by NEXTflex small RNA-Seq kit v3 (Perkin Elmer)). The trimmed reads were quality checked using FastQC v0.11.9 and MultiQC v1.12. The trimmed reads were then processed using the miRDeep2 analysis workflow. The sequences were aligned to *Bos taurus* reference genome ARS-UCD 1.2 using the mapper.pl module. The reads were further aligned with *Bos taurus* precursor to extract mature miRNAs from miRBase v22.1. The reads with size greater than 18 nt were mapped to generate read counts per sample. After generating the read counts per sample, the mature miRNAs not expressed in all the samples were filtered out. The reads were then transformed to counts per million (CPM) using edgeR [6], and the miRNAs with CPM < 1 in 50% of the samples were not considered for further analysis. For the differential expression analysis, the filtered raw counts were used in DESeq2 v1.26.0 [7]. The pregnancy status (pregnant to AI or non-pregnant) was considered for the design model used on the DESeq2 R-package. The differentially expressed miRNAs with *p-value* ≤ 0.05 and absolute (log2 fold change) ≥ 0.5 were considered significant.

## Ethics Statements

All procedures involving animals were approved by Institutional Animal Care and Use Committee (IACUC) at Auburn University and the guide for the Care and Use of Laboratory Animals (IACUC protocol number 2015-2786 and 2019-3591).

## CRediT authorship contribution statement

**Priyanka Banerjee:** Conceptualization, Methodology, Software, Data curation, Writing – original draft, Writing – review & editing. **Wellison J.S. Diniz:** Methodology, Data curation, Writing – review & editing. **Paul W. Dyce:** Conceptualization, Methodology, Supervision, Writing – review & editing.

## Data Availability

miRNA profiling from peripheral white blood cells in fertile and subfertile beef heifers at weaning (Original data) (NCBI GEO).
